# Supplementary material for: Resolving the full spectrum of human genome variation using Linked-Reads
Source: Genome Res. 2019 Apr;29(4):635–45. doi: 10.1101/gr.234443.118 (PMC6442396; doi:10.1101/gr.234443.118)
Supplement: Supplemental Material [file supp_gr.234443.118_Supplemental_longranger-2.2.zip › longranger-2.2.tar.bz2!/lr2.2-public/lib/stan/cmdstan/stan/doc/api/html/poisson_8hpp_source.html]

Stan: src/stan/prob/distributions/univariate/discrete/poisson.hpp Source File


|  |  |
| --- | --- |
|  | Stan  2.5.0  probability, sampling & optimization |


- Main Page
- Related Pages
- Namespaces
- Classes
- Files

- File List
- File Members

All Classes Namespaces Files Functions Variables Typedefs Enumerations Enumerator Friends Macros Pages

- src
- stan
- prob
- distributions
- univariate
- discrete

poisson.hpp

Go to the documentation of this file.

1 #ifndef STAN\_\_PROB\_\_DISTRIBUTIONS\_\_UNIVARIATE\_\_DISCRETE\_\_POISSON\_HPP

2 #define STAN\_\_PROB\_\_DISTRIBUTIONS\_\_UNIVARIATE\_\_DISCRETE\_\_POISSON\_HPP

3

4 #include <boost/random/poisson\_distribution.hpp>

5 #include <boost/random/variate\_generator.hpp>

6

7 #include <limits>

8 #include <boost/math/special\_functions/fpclassify.hpp>

9

10 #include <stan/agrad/partials\_vari.hpp>

11 #include <stan/math/error\_handling.hpp>

12 #include <stan/math/constants.hpp>

13 #include <stan/math/functions/multiply\_log.hpp>

14 #include <stan/math/functions/value\_of.hpp>

15 #include <stan/meta/traits.hpp>

16 #include <stan/prob/traits.hpp>

17 #include <stan/prob/constants.hpp>

18

19 namespace stan {

20

21  namespace prob {

22

23  // Poisson(n|lambda) [lambda > 0; n >= 0]

24  template <bool propto,

25  typename T\_n, typename T\_rate>

26  typename return\_type<T\_rate>::type

27  poisson\_log(const T\_n& n, const T\_rate& lambda) {

28

29  static const char\* function = "stan::prob::poisson\_log(%1%)";

30

31  using boost::math::lgamma;

32  using stan::math::check\_consistent\_sizes;

33  using stan::math::check\_not\_nan;

34  using stan::math::check\_nonnegative;

35  using stan::prob::include\_summand;

36  using stan::math::value\_of;

37

38  // check if any vectors are zero length

39  if (!(stan::length(n)

40  && stan::length(lambda)))

41  return 0.0;

42

43  // set up return value accumulator

44  double logp(0.0);

45

46  // validate args

47  check\_nonnegative(function, n, "Random variable", &logp);

48  check\_not\_nan(function, lambda,

49  "Rate parameter", &logp);

50  check\_nonnegative(function, lambda,

51  "Rate parameter", &logp);

52  check\_consistent\_sizes(function,

53  n,lambda,

54  "Random variable","Rate parameter",

55  &logp);

56

57  // check if no variables are involved and prop-to

58  if (!include\_summand<propto,T\_rate>::value)

59  return 0.0;

60

61  // set up expression templates wrapping scalars/vecs into vector views

62  VectorView<const T\_n> n\_vec(n);

63  VectorView<const T\_rate> lambda\_vec(lambda);

64  size\_t size = max\_size(n, lambda);

65

66  for (size\_t i = 0; i < size; i++)

67  if (boost::math::isinf(lambda\_vec[i]))

68  return LOG\_ZERO;

69  for (size\_t i = 0; i < size; i++)

70  if (lambda\_vec[i] == 0 && n\_vec[i] != 0)

71  return LOG\_ZERO;

72

73  // return accumulator with gradients

74  agrad::OperandsAndPartials<T\_rate> operands\_and\_partials(lambda);

75

76  using stan::math::multiply\_log;

77  for (size\_t i = 0; i < size; i++) {

78  if (!(lambda\_vec[i] == 0 && n\_vec[i] == 0)) {

79  if (include\_summand<propto>::value)

80  logp -= lgamma(n\_vec[i] + 1.0);

81  if (include\_summand<propto,T\_rate>::value)

82  logp += multiply\_log(n\_vec[i], value\_of(lambda\_vec[i]))

83  - value\_of(lambda\_vec[i]);

84  }

85

86  // gradients

87  if (!is\_constant\_struct<T\_rate>::value)

88  operands\_and\_partials.d\_x1[i]

89  += n\_vec[i] / value\_of(lambda\_vec[i]) - 1.0;

90

91  }

92

93

94  return operands\_and\_partials.to\_var(logp);

95  }

96

97  template <typename T\_n,

98  typename T\_rate>

99  inline

100  typename return\_type<T\_rate>::type

101  poisson\_log(const T\_n& n, const T\_rate& lambda) {

102  return poisson\_log<false>(n,lambda);

103  }

104

105  // PoissonLog(n|alpha) [n >= 0] = Poisson(n|exp(alpha))

106  template <bool propto,

107  typename T\_n, typename T\_log\_rate>

108  typename return\_type<T\_log\_rate>::type

109  poisson\_log\_log(const T\_n& n, const T\_log\_rate& alpha) {

110

111  static const char\* function = "stan::prob::poisson\_log\_log(%1%)";

112

113  using boost::math::lgamma;

114  using stan::math::check\_not\_nan;

115  using stan::math::check\_nonnegative;

116  using stan::math::value\_of;

117  using stan::math::check\_consistent\_sizes;

118  using stan::prob::include\_summand;

119  using std::exp;

120

121  // check if any vectors are zero length

122  if (!(stan::length(n)

123  && stan::length(alpha)))

124  return 0.0;

125

126  // set up return value accumulator

127  double logp(0.0);

128

129  // validate args

130  check\_nonnegative(function, n, "Random variable", &logp);

131  check\_not\_nan(function, alpha,

132  "Log rate parameter", &logp);

133  check\_consistent\_sizes(function,

134  n,alpha,

135  "Random variable","Log rate parameter",

136  &logp);

137

138  // check if no variables are involved and prop-to

139  if (!include\_summand<propto,T\_log\_rate>::value)

140  return 0.0;

141

142  // set up expression templates wrapping scalars/vecs into vector views

143  VectorView<const T\_n> n\_vec(n);

144  VectorView<const T\_log\_rate> alpha\_vec(alpha);

145  size\_t size = max\_size(n, alpha);

146

147  // FIXME: first loop size of alpha\_vec, second loop if-ed for size==1

148  for (size\_t i = 0; i < size; i++)

149  if (std::numeric\_limits<double>::infinity() == alpha\_vec[i])

150  return LOG\_ZERO;

151  for (size\_t i = 0; i < size; i++)

152  if (-std::numeric\_limits<double>::infinity() == alpha\_vec[i]

153  && n\_vec[i] != 0)

154  return LOG\_ZERO;

155

156  // return accumulator with gradients

157  agrad::OperandsAndPartials<T\_log\_rate> operands\_and\_partials(alpha);

158

159  // FIXME: cache value\_of for alpha\_vec? faster if only one?

160  DoubleVectorView<include\_summand<propto,T\_log\_rate>::value,

161  is\_vector<T\_log\_rate>::value>

162  exp\_alpha(length(alpha));

163  for (size\_t i = 0; i < length(alpha); i++)

164  if (include\_summand<propto,T\_log\_rate>::value)

165  exp\_alpha[i] = exp(value\_of(alpha\_vec[i]));

166  using stan::math::multiply\_log;

167  for (size\_t i = 0; i < size; i++) {

168  if (!(alpha\_vec[i] == -std::numeric\_limits<double>::infinity()

169  && n\_vec[i] == 0)) {

170  if (include\_summand<propto>::value)

171  logp -= lgamma(n\_vec[i] + 1.0);

172  if (include\_summand<propto,T\_log\_rate>::value)

173  logp += n\_vec[i] \* value\_of(alpha\_vec[i]) - exp\_alpha[i];

174  }

175

176  // gradients

177  if (!is\_constant\_struct<T\_log\_rate>::value)

178  operands\_and\_partials.d\_x1[i] += n\_vec[i] - exp\_alpha[i];

179  }

180  return operands\_and\_partials.to\_var(logp);

181  }

182

183  template <typename T\_n,

184  typename T\_log\_rate>

185  inline

186  typename return\_type<T\_log\_rate>::type

187  poisson\_log\_log(const T\_n& n, const T\_log\_rate& alpha) {

188  return poisson\_log\_log<false>(n,alpha);

189  }

190

191  // Poisson CDF

192  template <typename T\_n, typename T\_rate>

193  typename return\_type<T\_rate>::type

194  poisson\_cdf(const T\_n& n, const T\_rate& lambda) {

195  static const char\* function = "stan::prob::poisson\_cdf(%1%)";

196

197  using stan::math::check\_not\_nan;

198  using stan::math::check\_nonnegative;

199  using stan::math::value\_of;

200  using stan::math::check\_consistent\_sizes;

201

202  // Ensure non-zero argument slengths

203  if (!(stan::length(n) && stan::length(lambda)))

204  return 1.0;

205

206  double P(1.0);

207

208  // Validate arguments

209  check\_not\_nan(function, lambda, "Rate parameter", &P);

210  check\_nonnegative(function, lambda, "Rate parameter", &P);

211  check\_consistent\_sizes(function, n,lambda,

212  "Random variable","Rate parameter",

213  &P);

214

215  // Wrap arguments into vector views

216  VectorView<const T\_n> n\_vec(n);

217  VectorView<const T\_rate> lambda\_vec(lambda);

218  size\_t size = max\_size(n, lambda);

219

220  // Compute vectorized CDF and gradient

221  using stan::math::value\_of;

222  using boost::math::gamma\_p\_derivative;

223  using boost::math::gamma\_q;

224

225  agrad::OperandsAndPartials<T\_rate> operands\_and\_partials(lambda);

226

227  // Explicit return for extreme values

228  // The gradients are technically ill-defined, but treated as zero

229  for (size\_t i = 0; i < stan::length(n); i++) {

230  if (value\_of(n\_vec[i]) < 0)

231  return operands\_and\_partials.to\_var(0.0);

232  }

233

234  for (size\_t i = 0; i < size; i++) {

235  // Explicit results for extreme values

236  // The gradients are technically ill-defined, but treated as zero

237  if (value\_of(n\_vec[i]) == std::numeric\_limits<double>::infinity())

238  continue;

239

240  const double n\_dbl = value\_of(n\_vec[i]);

241  const double lambda\_dbl = value\_of(lambda\_vec[i]);

242  const double Pi = gamma\_q(n\_dbl+1, lambda\_dbl);

243

244  P \*= Pi;

245

246  if (!is\_constant\_struct<T\_rate>::value)

247  operands\_and\_partials.d\_x1[i]

248  -= gamma\_p\_derivative(n\_dbl + 1, lambda\_dbl) / Pi;

249  }

250

251  if (!is\_constant\_struct<T\_rate>::value)

252  for(size\_t i = 0; i < stan::length(lambda); ++i)

253  operands\_and\_partials.d\_x1[i] \*= P;

254

255  return operands\_and\_partials.to\_var(P);

256  }

257

258  template <typename T\_n, typename T\_rate>

259  typename return\_type<T\_rate>::type

260  poisson\_cdf\_log(const T\_n& n, const T\_rate& lambda) {

261  static const char\* function = "stan::prob::poisson\_cdf\_log(%1%)";

262

263  using stan::math::check\_not\_nan;

264  using stan::math::check\_nonnegative;

265  using stan::math::value\_of;

266  using stan::math::check\_consistent\_sizes;

267

268  // Ensure non-zero argument slengths

269  if (!(stan::length(n) && stan::length(lambda)))

270  return 0.0;

271

272  double P(0.0);

273

274  // Validate arguments

275  check\_not\_nan(function, lambda, "Rate parameter", &P);

276  check\_nonnegative(function, lambda, "Rate parameter", &P);

277  check\_consistent\_sizes(function, n,lambda,

278  "Random variable","Rate parameter",

279  &P);

280

281  // Wrap arguments into vector views

282  VectorView<const T\_n> n\_vec(n);

283  VectorView<const T\_rate> lambda\_vec(lambda);

284  size\_t size = max\_size(n, lambda);

285

286  // Compute vectorized cdf\_log and gradient

287  using stan::math::value\_of;

288  using boost::math::gamma\_p\_derivative;

289  using boost::math::gamma\_q;

290

291  agrad::OperandsAndPartials<T\_rate> operands\_and\_partials(lambda);

292

293  // Explicit return for extreme values

294  // The gradients are technically ill-defined, but treated as neg infinity

295  for (size\_t i = 0; i < stan::length(n); i++) {

296  if (value\_of(n\_vec[i]) < 0)

297  return operands\_and\_partials.to\_var(stan::math::negative\_infinity());

298  }

299

300  for (size\_t i = 0; i < size; i++) {

301  // Explicit results for extreme values

302  // The gradients are technically ill-defined, but treated as zero

303  if (value\_of(n\_vec[i]) == std::numeric\_limits<double>::infinity())

304  continue;

305

306  const double n\_dbl = value\_of(n\_vec[i]);

307  const double lambda\_dbl = value\_of(lambda\_vec[i]);

308  const double Pi = gamma\_q(n\_dbl+1, lambda\_dbl);

309

310  P += log(Pi);

311

312  if (!is\_constant\_struct<T\_rate>::value)

313  operands\_and\_partials.d\_x1[i]

314  -= gamma\_p\_derivative(n\_dbl + 1, lambda\_dbl) / Pi;

315  }

316

317  return operands\_and\_partials.to\_var(P);

318  }

319

320  template <typename T\_n, typename T\_rate>

321  typename return\_type<T\_rate>::type

322  poisson\_ccdf\_log(const T\_n& n, const T\_rate& lambda) {

323  static const char\* function = "stan::prob::poisson\_ccdf\_log(%1%)";

324

325  using stan::math::check\_not\_nan;

326  using stan::math::check\_nonnegative;

327  using stan::math::value\_of;

328  using stan::math::check\_consistent\_sizes;

329

330  // Ensure non-zero argument slengths

331  if (!(stan::length(n) && stan::length(lambda)))

332  return 0.0;

333

334  double P(0.0);

335

336  // Validate arguments

337  check\_not\_nan(function, lambda, "Rate parameter", &P);

338  check\_nonnegative(function, lambda, "Rate parameter", &P);

339  check\_consistent\_sizes(function, n,lambda,

340  "Random variable","Rate parameter",

341  &P);

342

343  // Wrap arguments into vector views

344  VectorView<const T\_n> n\_vec(n);

345  VectorView<const T\_rate> lambda\_vec(lambda);

346  size\_t size = max\_size(n, lambda);

347

348  // Compute vectorized cdf\_log and gradient

349  using stan::math::value\_of;

350  using boost::math::gamma\_p\_derivative;

351  using boost::math::gamma\_q;

352

353  agrad::OperandsAndPartials<T\_rate> operands\_and\_partials(lambda);

354

355  // Explicit return for extreme values

356  // The gradients are technically ill-defined, but treated as neg infinity

357  for (size\_t i = 0; i < stan::length(n); i++) {

358  if (value\_of(n\_vec[i]) < 0)

359  return operands\_and\_partials.to\_var(0.0);

360  }

361

362  for (size\_t i = 0; i < size; i++) {

363  // Explicit results for extreme values

364  // The gradients are technically ill-defined, but treated as zero

365  if (value\_of(n\_vec[i]) == std::numeric\_limits<double>::infinity())

366  return operands\_and\_partials.to\_var(stan::math::negative\_infinity());

367

368  const double n\_dbl = value\_of(n\_vec[i]);

369  const double lambda\_dbl = value\_of(lambda\_vec[i]);

370  const double Pi = 1.0 - gamma\_q(n\_dbl+1, lambda\_dbl);

371

372  P += log(Pi);

373

374  if (!is\_constant\_struct<T\_rate>::value)

375  operands\_and\_partials.d\_x1[i]

376  += gamma\_p\_derivative(n\_dbl + 1, lambda\_dbl) / Pi;

377  }

378

379  return operands\_and\_partials.to\_var(P);

380  }

381

382  template <class RNG>

383  inline int

384  poisson\_rng(const double lambda,

385  RNG& rng) {

386  using boost::variate\_generator;

387  using boost::random::poisson\_distribution;

388

389  static const char\* function = "stan::prob::poisson\_rng(%1%)";

390

391  using stan::math::check\_not\_nan;

392  using stan::math::check\_nonnegative;

393

394  check\_not\_nan(function, lambda,

395  "Rate parameter", (double\*)0);

396  check\_nonnegative(function, lambda,

397  "Rate parameter", (double\*)0);

398

399  variate\_generator<RNG&, poisson\_distribution<> >

400  poisson\_rng(rng, poisson\_distribution<>(lambda));

401  return poisson\_rng();

402  }

403

404  }

405 }

406 #endif

multiply\_log.hpp

traits.hpp

stan::agrad::OperandsAndPartials::to\_var

T\_return\_type to\_var(double logp)

**Definition:** partials\_vari.hpp:138

error\_handling.hpp

stan::math::multiply\_log

boost::math::tools::promote\_args< T\_a, T\_b >::type multiply\_log(const T\_a a, const T\_b b)

Calculated the value of the first argument times log of the second argument while behaving properly w...

**Definition:** multiply\_log.hpp:51

stan::length

size\_t length(const T &)

**Definition:** traits.hpp:159

stan::DoubleVectorView

DoubleVectorView allocates double values to be used as intermediate values.

**Definition:** traits.hpp:358

partials\_vari.hpp

stan::agrad::value\_of

T value\_of(const fvar< T > &v)

Return the value of the specified variable.

**Definition:** value\_of.hpp:16

constants.hpp

stan::is\_vector

**Definition:** traits.hpp:83

stan::prob::poisson\_log

return\_type< T\_rate >::type poisson\_log(const T\_n &n, const T\_rate &lambda)

**Definition:** poisson.hpp:27

stan::agrad::lgamma

fvar< T > lgamma(const fvar< T > &x)

**Definition:** lgamma.hpp:15

stan::agrad::OperandsAndPartials

A variable implementation that stores operands and derivatives with respect to the variable...

**Definition:** partials\_vari.hpp:76

traits.hpp

stan::return\_type::type

boost::math::tools::promote\_args< typename scalar\_type< T1 >::type, typename scalar\_type< T2 >::type, typename scalar\_type< T3 >::type, typename scalar\_type< T4 >::type, typename scalar\_type< T5 >::type, typename scalar\_type< T6 >::type >::type type

**Definition:** traits.hpp:406

stan::is\_constant\_struct

Metaprogram to determine if a type has a base scalar type that can be assigned to type double...

**Definition:** traits.hpp:57

stan::math::value\_of

double value\_of(const T x)

Return the value of the specified scalar argument converted to a double value.

**Definition:** value\_of.hpp:24

boost::math::isinf

bool isinf(const stan::agrad::var &v)

Checks if the given number is infinite.

**Definition:** boost\_fpclassify.hpp:54

stan::prob::include\_summand

Template metaprogram to calculate whether a summand needs to be included in a proportional (log) prob...

**Definition:** traits.hpp:35

stan::prob::poisson\_cdf\_log

return\_type< T\_rate >::type poisson\_cdf\_log(const T\_n &n, const T\_rate &lambda)

**Definition:** poisson.hpp:260

stan::prob::poisson\_rng

int poisson\_rng(const double lambda, RNG &rng)

**Definition:** poisson.hpp:384

stan::prob::poisson\_log\_log

return\_type< T\_log\_rate >::type poisson\_log\_log(const T\_n &n, const T\_log\_rate &alpha)

**Definition:** poisson.hpp:109

stan::agrad::gamma\_q

fvar< T > gamma\_q(const fvar< T > &x1, const fvar< T > &x2)

**Definition:** gamma\_q.hpp:15

stan::math::check\_nonnegative

bool check\_nonnegative(const char \*function, const T\_y &y, const char \*name, T\_result \*result)

**Definition:** check\_nonnegative.hpp:52

stan::math::check\_consistent\_sizes

bool check\_consistent\_sizes(const char \*function, const T1 &x1, const T2 &x2, const char \*name1, const char \*name2, T\_result \*result)

**Definition:** check\_consistent\_sizes.hpp:13

stan::max\_size

size\_t max\_size(const T1 &x1, const T2 &x2)

**Definition:** traits.hpp:191

stan::math::check\_not\_nan

bool check\_not\_nan(const char \*function, const T\_y &y, const char \*name, T\_result \*result)

Checks if the variable y is nan.

**Definition:** check\_not\_nan.hpp:56

stan::agrad::multiply\_log

fvar< T > multiply\_log(const fvar< T > &x1, const fvar< T > &x2)

**Definition:** multiply\_log.hpp:15

stan::math::size

int size(const std::vector< T > &x)

**Definition:** size.hpp:11

value\_of.hpp

stan::prob::poisson\_ccdf\_log

return\_type< T\_rate >::type poisson\_ccdf\_log(const T\_n &n, const T\_rate &lambda)

**Definition:** poisson.hpp:322

stan::prob::poisson\_cdf

return\_type< T\_rate >::type poisson\_cdf(const T\_n &n, const T\_rate &lambda)

**Definition:** poisson.hpp:194

stan::agrad::OperandsAndPartials::d\_x1

VectorView< double \*, is\_vector< T1 >::value, is\_constant\_struct< T1 >::value > d\_x1

**Definition:** partials\_vari.hpp:82

constants.hpp

stan::agrad::log

fvar< T > log(const fvar< T > &x)

**Definition:** log.hpp:15

stan::VectorView

VectorView is a template metaprogram that takes its argument and allows it to be used like a vector...

**Definition:** traits.hpp:275

stan::agrad::exp

fvar< T > exp(const fvar< T > &x)

**Definition:** exp.hpp:16

stan::math::negative\_infinity

double negative\_infinity()

Return negative infinity.

**Definition:** constants.hpp:123


---

[ Stan Home Page ]

*© 2011–2014,
Stan Development Team.*
